# Quadrupedal Robot Locomotion: A Biologically Inspired Approach and Its Hardware Implementation

**DOI:** 10.1155/2016/5615618

**Published:** 2016-06-29

**Authors:** A. Espinal, H. Rostro-Gonzalez, M. Carpio, E. I. Guerra-Hernandez, M. Ornelas-Rodriguez, H. J. Puga-Soberanes, M. A. Sotelo-Figueroa, P. Melin

**Affiliations:** ^1^Division of Postgraduate Studies and Research, Leon Institute of Technology, 37290 Leon, GTO, Mexico; ^2^Department of Electronics, DICIS, University of Guanajuato, 36885 Salamanca, GTO, Mexico; ^3^Department of Organizational Studies, DCEA, University of Guanajuato, 36250 Guanajuato, GTO, Mexico; ^4^Division of Postgraduate Studies and Research, Tijuana Institute of Technology, 22414 Tijuana, BC, Mexico

## Abstract

A bioinspired locomotion system for a quadruped robot is presented. Locomotion is achieved by a spiking neural network (SNN) that acts as a Central Pattern Generator (CPG) producing different locomotion patterns represented by their raster plots. To generate these patterns, the SNN is configured with specific parameters (synaptic weights and topologies), which were estimated by a metaheuristic method based on Christiansen Grammar Evolution (CGE). The system has been implemented and validated on two robot platforms; firstly, we tested our system on a quadruped robot and, secondly, on a hexapod one. In this last one, we simulated the case where two legs of the hexapod were amputated and its locomotion mechanism has been changed. For the quadruped robot, the control is performed by the spiking neural network implemented on an Arduino board with 35% of resource usage. In the hexapod robot, we used Spartan 6 FPGA board with only 3% of resource usage. Numerical results show the effectiveness of the proposed system in both cases.

## 1. Introduction

Autonomous robot locomotion is a problem that has been partially solved. To deal with the inherent locomotion problems, different mechanisms have been implemented. Some of them have been implemented through wheels due to simplicity. However, this approach presents disadvantages related to the environment surface. Hence, previous research work has addressed the feasibility of implementing locomotion mechanism based on legs. Legged locomotion results from complex, high-dimensional, nonlinear, dynamically coupled interactions between an organism and its environment. Fortunately, models have been proposed to resolve the redundancy of multiple legs, joints, and muscles by seeking synergies and symmetries [1]. In the literature, there are two main approaches for the design of locomotion control systems such as kinematic and dynamic mathematical models and biologically inspired approaches [2]. In the first one, to move a leg in a desired trajectory, the joint angles are calculated in advance, by using a mathematical model that incorporates both robot and environment parameters, to produce a sequence of actions algorithmically scheduled [3]; these kinds of algorithms can be too complex and unable to be used in dynamic environments with real time response restrictions. The second approach uses bioinspired principles found in nature. From an engineering viewpoint, the main reason for the great interest in bioinspired approaches is the fact that they provide suitable solutions for the design of efficient walking robots. Usually, bioinspired solutions use common principles found in a large variety of animals. Applications of these principles are possible since major advances have been made by biologists in understanding animal locomotion, and at the same time artificial locomotion systems are interesting topics of study, in particular robotics, since they are a good realistic way to verify a hypothesis regarding the biological model and a good source for new ideas [4].

Biologists often assume that vertebrate locomotion is controlled by a Central Pattern Generator (CPG) capable of producing rhythmic patterns or gaits. CPGs have the ability to automatically generate complex control signals for the coordination of muscles during rhythmic movements, such as walking, running, swimming, and flying [5, 6]. CPGs have been used to control a variety of different types of robots and different modes of locomotion. For example, CPG models have been used with hexapod and octopod robots inspired by insect locomotion, quadruped robots inspired by vertebrates, such as horse, biped robots inspired by humans, and other kinds of robots inspired by reptiles, such as snakes. Different levels of abstraction have been used to model CPGs; depending on the phenomena under study the CPG can be designed from detailed biophysical models to abstract systems of coupled oscillators [2, 7]. CPGs present several interesting properties including distributed control, the ability to deal with redundancies, robustness against perturbations, and feedback loops allowing modulation of locomotion in unknown environments by simple control signal. These properties, when transferred to mathematical models, make CPGs interesting building blocks for locomotion controllers in mobile robots [7].

CPGs have been implemented using general purpose processor providing high accuracy and flexibility but those systems consume relatively high power and occupy a large area, restricting their utility in embedded applications. Additionally, in these processors each task gets time on the CPU regardless of its priority and even the most time-critical application can be suspended for some routine maintenance; these two features have a considerable effect on performance and real time response cannot be ensured [9]. These processors have begun to inherit high-performance techniques from their desktop counterparts, such as pipelining, caches, dynamic branch prediction, and multithreading. Unfortunately, even when these techniques offer a good solution in software, their performance cannot be analytically bounded, so when a task will be executed cannot be determined accurately. As a consequence, CPG dedicated hardware implementation, both analog and digital, has received more attention. On one hand, analog circuits have been already proposed, being computational and power efficient, but they usually lack flexibility and they involve large design cycles. Although there is efficient locomotion control based on CPGs, few works have focused on adopting the technology to fully practical embedded implementation with the ability to be scalable or reusable in different robots morphologies [10–12]. Recent developments in embedded controller technology have yielded very sophisticated computing devices in relatively small and easily programmed modules. These technologies are of low cost, power efficient, and adaptive, which might greatly benefit from custom hardware architectures. These architectures can be an alternative to implement robot control schemes that counterbalance the fully analog and digital drawbacks by providing custom efficient hardware attached to embedded processors in a single chip (SoC).

Recently, FPGA (Field Programmable Gate Array) technology has improved in density up to the point that it is feasible to implement large scale bioinspired systems on a single FPGA device. Many interesting bioinspired systems such as locomotion control based on CPGs can be implemented using this technology [13]. FPGAs offer a computational architecture that is well suited for algorithms that require massive parallelism of fine-grained computational units. The inherent parallelism of the logic resources, as well as the availability of hard cores (such as multipliers, large distributed Random Access Memory (RAM) blocks, Digital Signal Processing (DSP), and slices) on the FPGA, allows a considerable computation throughput even at sub-500 MHz clock rates. Although CPGs might not be highly computationally demanding, autonomous robot locomotion needs additional modules to carry out interaction tasks with the environment, and, in general, these tasks are complex to be achieved in embedded general purpose processors by themselves. FPGA implementation can provide flexibility and lower latency and real time responsiveness compared to software-based embedded systems.

## 2. Materials and Methods

### 2.1. The Spiking Neuron Model

In its simplest form, the evolution of the membrane potential of the integrate-and-fire spiking neuron model is described by the following equations (see [14, 15] for more details about the derivation):

(1) Vk=γVk−11−Zk−1+∑j=1NWjZjk−1+Iextk−1,

(2) Z=1if V≥θfiring0otherwise,

where *V*[*k*] represents the membrane potential of a neuron at a discrete time *k*. *γ* ∈ [0,1[ defines the leak rate. The firing state is given by the term *Z* in (2). *N* is the number of presynaptic neurons. *W* is the matrix of synaptic weights. Finally, *I* ^ext^ represents an external stimulus.

When *V*[*k*] reaches a given threshold *θ*, then a spike occurs in *Z*[*k*] and the neuron is reset by the term (1 − *Z*_*i*_[*k*]) in (1).

### 2.2. The Locomotion System

**Figure 1:**
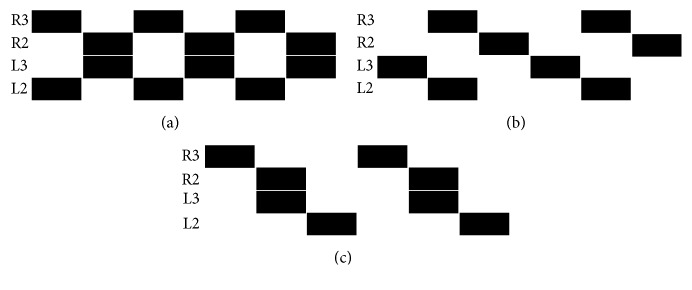
Schematic drawing of different stereotypic quadrupedal walking patterns. In trot, two diagonal legs swing in synchrony (a). In walk, synchronous swing of a diagonal pair of legs is followed by two single leg swing phases (b, c). Black bars indicate leg swing and R and L correspond to right and left sides, respectively [8].

The locomotion system is a spiking neural network (SNN) that acts as a Central Pattern Generator (CPG) [2]. That is, the SNN is able to generate different periodic patterns (locomotion gaits), such as those observed for interleg coordination in free-walking adult stick insects and shown in Figure 1 [8]. Such patterns can be represented as spike trains of *N* neurons (8 for both robots, quadruped and hexapod), which are estimated from the following equation:

(3) Vik=γVik−11−Zik−1+∑j=1NWijZjk−1+Iiextk−1,

and the spiking activity (spike train) of the whole network is defined by *Z* as indicated in (2).

The synaptic weights (*W*) are represented as *N* × *N* matrix, which defines a specific topology for the spiking neural network and it is determined by the methodology described in Section 2.3. Once these parameters have been estimated they are used in (3) to generate the desired locomotion gait. For a practical reason, the value of *γ* (leakage parameter) has been set to 0.5; this is due to the fact that such value is a power of two (2^−1^), which is highly suitable for hardware implementation (*binary* operations). The CPGs can produce rhythmic signals without afferent sensory information, and for this reason the SNN does not require exogenous inputs; that is, *I* ^ext^ has been set to zero. Finally, the initial conditions for *Z* and *V* correspond to the values at the time *k* − 1 of the desired locomotion gait.

**Figure 2:**
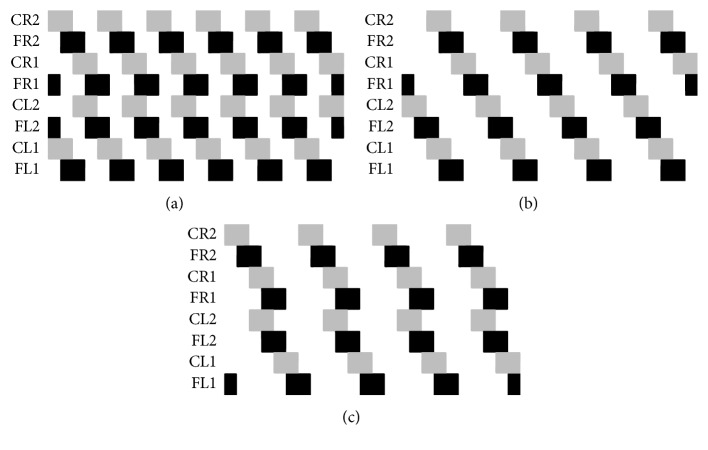
Modified locomotion patterns for the legged robots.

In this research, the locomotion patterns shown in Figure 1 have been modified in order to match with the robot structure. To be more specific, the quadruped and hexapod robots have 12 and 18 Degrees of Freedom (DOFs), respectively, with 3 servomotors (DOFs) per leg, which correspond to femur, tibia, and coxa. However, for locomotion we only need to control 2 of them, femur and coxa (see Figure 5). The servomotor for the tibia only needs to be energised but not controlled. This is due to the fact that the tibia is the weight-bearing part of the robot. Thus, the improved locomotion patterns are shown in Figure 2.

### 2.3. Parameter Estimation

The parameter estimation of Central Pattern Generators (CPGs) is generally a difficult task; on it depends the spiking neural network's (SNN) capability to periodically replicate a set of rhythmic signals [2]. In this work, the parameter estimation (synaptic weights and connections) of SNNs follows a divide-and-conquer workflow based on an evolutionary approach. Different evolutionary approaches to deal with design and tuning parameters of Artificial Neural Networks have been proposed, for example, weight tuning, topology definition, learning rule optimization, and combination of them. Besides, these evolutionary approaches can search directly or indirectly over the search space according to the representation of candidate solutions (see [16] for a detailed review). Particularly, to tackle the parameter estimation in CPG-based systems driven by evolutionary algorithms, most works use evolutionary algorithms to modulate synaptic parameters of prefixed network topologies. In [17], a Genetic Algorithm for tunning the parameters of CPG designed for the locomotion of both terrestrial and aquatic gaits of a virtual salamander was successfully implemented. However, the most related work to this research is presented in [18], where neural networks are developed and designed by means of Genetic Programming (GP) to make a virtual fish swim, and this work reports the feasibility of using GP to develop CPGs; however, those designs were not implemented on a real robot.

The parameter estimation method deals with an optimization problem, where the search space is formed by all weighted connectivity configurations for a graph with *N* nodes; hence, the matrix *W* in (3) defines both the topology and synaptic weights of a SNN; it can be considered as the adjacency transpose matrix of a weighted directed graph. The parameter estimation method divides the design of a SNN into individual connectivity designs for each spiking neuron, instead of designing and training a SNN as a whole. Each connectivity design of a neuron is carried out by an evolutionary approach that represents solutions indirectly. The purpose of the evolutionary method is to find out a set of weighted connections for a neuron to periodically replicate a target rhythmic pattern according to a desired gait. The connectivity configuration of each neuron is represented by words, and (4) shows the syntax for connectivity words, where the first part (before the sign “:”) indicates the number of presynaptic connections of the current neuron and the second part (after the sign “:”) indicates the indexes and synaptic weights of each presynaptic neuron:

(4) n︸synaptic connections:id1st,weight1st︷1st configured synapse⋯idnth,weightnth︷nth configured synapse.

The words in (4) besides syntactic correctness require ensuring semantic criteria related to the matrix *W* in (3) such as the following: the number of presynaptic neurons must be bounded 1 ≤ *n* ≤ *N* and the indexes of the presynaptic neurons must be different (id_1_ ≠ id_2_ ≠ ⋯≠id_*n*th_) to avoid multiples ties or loops from the same presynaptic neuron to the current neuron being designed (or multiple values into a single cell from the matrix *W*).

To obtain solutions that are syntactically and semantically correct such as in (4), the configuration for each neuron is carried out by Christiansen Grammar Evolution (CGE) [19] framework and requires the next three components:

(i) A Christiansen Grammar (CG) which reflects the syntactic and semantic requirements for the language of neuron connectivities. Several CG can be designed to cover these requirements, for example, a CG that removes neuron indexes when they were used (see the appendix).
(ii) A fitness function to set the quality of a candidate solution once it has been mapped from its genotypical form (string of numbers) to its phenotypical form (a spiking neuron connected to its presynaptic neurons). An important criterion to achieve that a specific neuron replicates an input rhythmic signal is the design of a fitness function to explore the search space of weighted connectivity configurations. In functional approximation, an alternate and explicit mathematical expression is constructed for the objective function [20], which in this case is unknown. In this work, the SPIKE-distance is used as basis of functional approximation for the fitness function. The SPIKE-distance is a parameter-free and timescale-adaptive measure for estimating the degree of synchrony between spike trains. In general, the distance is defined as a temporal average of the spike trains' dissimilarity profiles (see [21] for detailed definition). Here, the bivariate SPIKE-distance is used to measure the similarity between a target spike train (rhythmic signal) and the spike train generated after simulation by the phenotypical form of the candidate solution.
(iii) A search engine (metaheuristic algorithm) to drive the search of good solutions based on their quality. Here, a continuous Univariate Marginal Distribution Algorithm (UMDA_*c*_^*G*^) with elitism is used to evolve the connectivity designs as that is an easy-to-implement evolutionary algorithm (see [22] for implementation details).

The whole design process works as follows: a gait represented as a set of spike trains (rhythmic signals) is given as input. Next, each spike train (rhythmic signal) is treated individually for being designed, and all the spike trains except the targeted one are set as available as activity of feasible presynaptic neurons and the initial state of the current designed neuron is set according to the input gait, if at time *k* = 0 the neuron should fire or should not. Once the required configurations are done the CGE framework evolves candidate solutions for connectivity until an expected error is achieved (this depends on the fitness function being used); after the gait's replication is achieved, the current neuron connectivity design is stored and the process continues until achieving the correct replication of *N* spike trains from the input gait. Finally, all *N* individual neuron connectivity designs are integrated into a SNN (see Algorithm 1).

**Figure 3:**
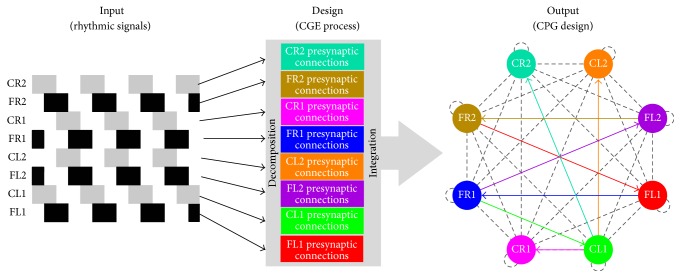
Schematic diagram of the CGE-based methodology for designing CPGs.

In Figure 3, a graphic workflow of the design method is presented, an input is decomposed into individual spike trains, and each neuron is configured to replicate a specific spike train. Later, all the individual designs are integrated into a SNN; the output is the CPG design, which is illustrated as a directed graph (weights have been omitted for clarity in the graphic), where each neuron configuration in the design part is associated with a color and its connectivity pattern can be visualised in the final graph (the final graph shows that the design does not use all available ties and loops; only the non-gray colored and continuous ones define the CPG topology).

### 2.4. Hardware

To validate the different configurations of the CPGs we have performed hardware implementation on dedicated hardware, such as Arduino (Microcontroller) and FPGA board (from OpalKelly) for high and low level implementation, respectively.

**Figure 4:**
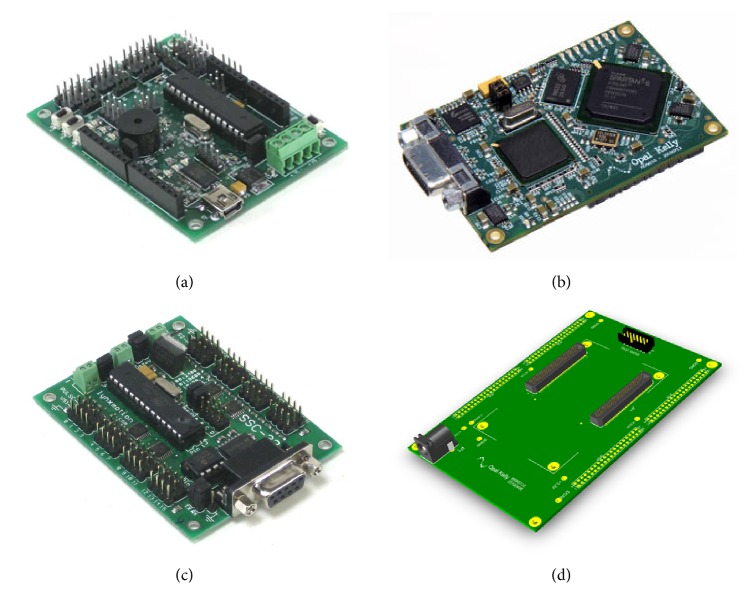
Hardware used in this research: (a) BotBoarduino, (b) FPGA, (c) servo controller SSC32, and (d) breakout board BRK6110.

The Arduino board used in this work is the BotBoarduino (Figure 4(a)), which is based on Atom Microcontrollers for Lynxmotion robots. It has an onboard speaker, three buttons and LEDs, a Sony PS2 controller port, a reset button, logic and servo power inputs, an I/O bus with 20 pins and power and ground, and a 5 vdc 1.5 amp regulator. Also, up to 18 servos can be plugged in directly.

In this work, we also considered the implementation on FPGA board in order to have better hardware conditions, such as more resources to implement complex designs, a hardware design language (HDL), low power consumption, reconfigurability, hardware parallelism, and very high processing speed. Specifically, in this work we use Spartan 6 XEM6310-LX45 board (Figure 4(b)) from the OpalKelly family (https://www.opalkelly.com/). This specific board has two more advantages: on one hand the dimensions, only 75 mm × 50 mm (highly suitable for our robots), and on the other hand a graphical interface for friendly interaction between the PC and the FPGA.

In both cases, a SSC-32 servo controller (Figure 4(c)) is used to handle servomotors in the robot. This is a servo controller with 32 channels of 1uS resolution servo control and a bidirectional communication.

To connect and control the servos through the FPGA, we also use a breakout board (BRK6110), which allows us an easy connection to high-density connectors on the XEM6110-LX45 by routing all signals to four 40-pin 2-mm headers (see Figure 4(d)).

**Figure 5:**
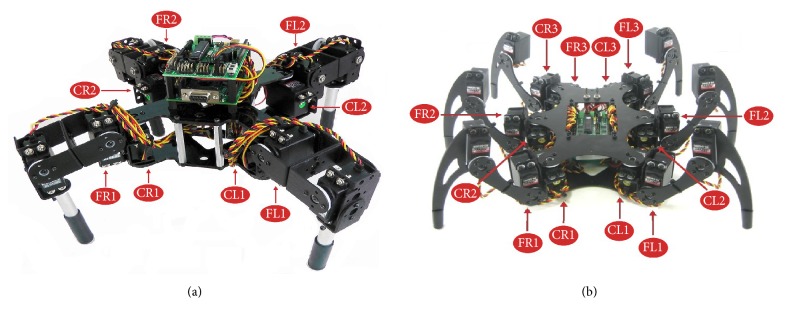
Neuronal configurations in the legged robots. (a) Quadruped and (b) hexapod robots. C and F indicate coxa and femur, respectively. L and R correspond to the side where the neurons are located in the robot (images from Lynxmotion website).

To validate the CPGs, we have used real quadruped and hexapod robots, such as those shown in Figure 5.

## 3. Results

Here, we present test results for the performance of the system. We first estimated the synaptic weights for three different gaits (walking, jogging, and running) of the SNNs by using Christiansen Grammar Evolution. For this, we performed three strategies based on three different SPIKE-distance-based fitness functions as follows:

- The Christiansen Grammar Evolution runs with a SPIKE-distance-based fitness function which has no restrictions on the number of synaptic connections.
- The Christiansen Grammar Evolution runs with a SPIKE-distance-based fitness function with restrictions on the number of synaptic connections; that is, we expect that only one presynaptic neuron can stimulate the postsynaptic neuron to reproduce its input signal.
- The Christiansen Grammar Evolution has a SPIKE-distance-based fitness configured to reproduce the three different locomotion patterns (gaits) with the same spiking neural network topology.

From these strategies, we obtained the different configurations for the spiking neural networks, which generate the locomotion patterns for the legged robots.

Equations (5), (6), and (7) correspond to the connectivity matrices for walking, jogging, and running gaits, respectively, generated by the Christian Grammar Evolution with no restrictions on the number of synaptic connections among the neurons. Similarly, (8), (9), and (10) correspond to the connectivity matrices for the same gaits but with restrictions on the number of synaptic connections. Finally, (11) corresponds to the connectivity matrix which can reproduce any of the three gaits; this is given when the third strategy is used with Christiansen Grammar Evolution.

**Figure 6:**
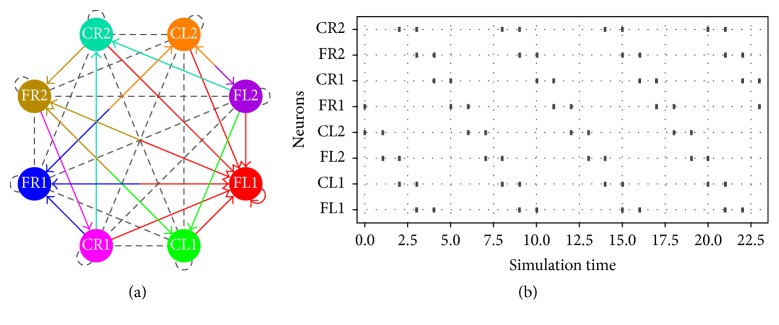
(a) Network topology and (b) raster plot for the walking gait.

**Figure 7:**
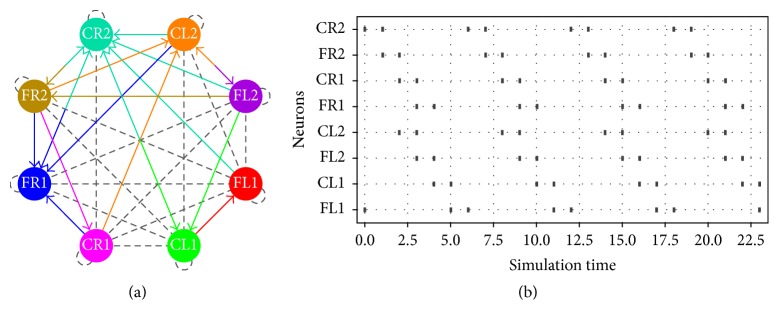
(a) Network topology and (b) raster plot for the jogging gait.

**Figure 8:**
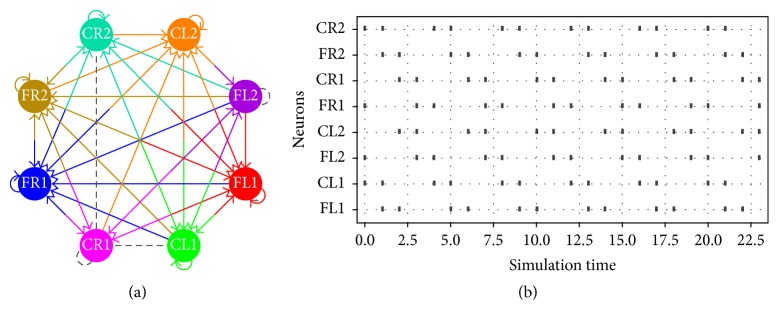
(a) Network topology and (b) raster plot for the running gait.

Besides, in Figures 6, 7, and 8, the schematic on the left side corresponds to the spiking neural networks topologies for walking, jogging, and running gaits, respectively. On the right side the raster plots (spiking activity) for the three locomotion patterns are shown:

(5) ww1=−542−92−732005000−200001000000−709000−300−3070000000080−4700000800400−200,

(6) wj1=050000000050000−30005000000−100−34000080−18−60000004000−50000467−25−70−60,

(7) wr1=−794−10−6−37−5−89500030−704080026−8−2−7−365−1−765−47−4480−20−407067−107−5−7−4−7−99030−41,

(8) ww2=0600000000100000000900000000200000000600100000000200000000600000,

(9) wj2=0900000000007000000400000000007000000700000000100000000980000000,

(10) wr2=0000000100003000000008000000005000000200400000000900000000600000.

In Figure 11, we show the network topologies when the parameter estimation presents restrictions on the number of synaptic connections. As we can observe, the connectivity map is clearer and easier to be implemented in hardware. The raster plots are exactly the same as those shown in Figures 6(b), 7(b), and 8(b).

In Figure 12, we present the most interesting implementation, because such network is able to generate the three locomotion patterns presented in this work.

**Figure 9:**
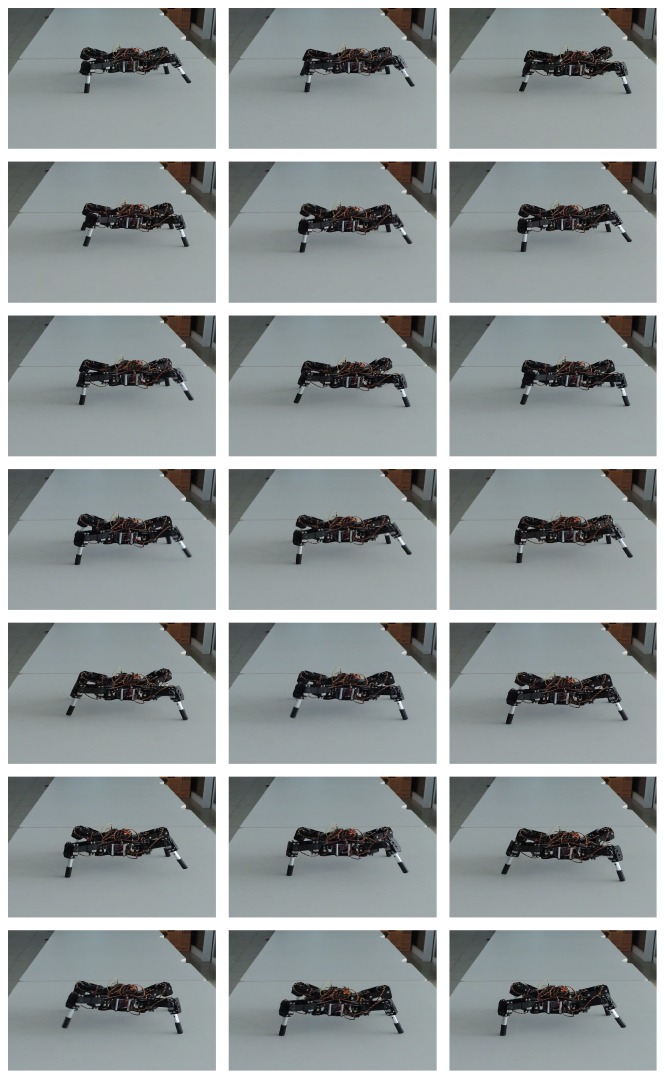
Real time simulation on a quadruped robot.

**Figure 10:**
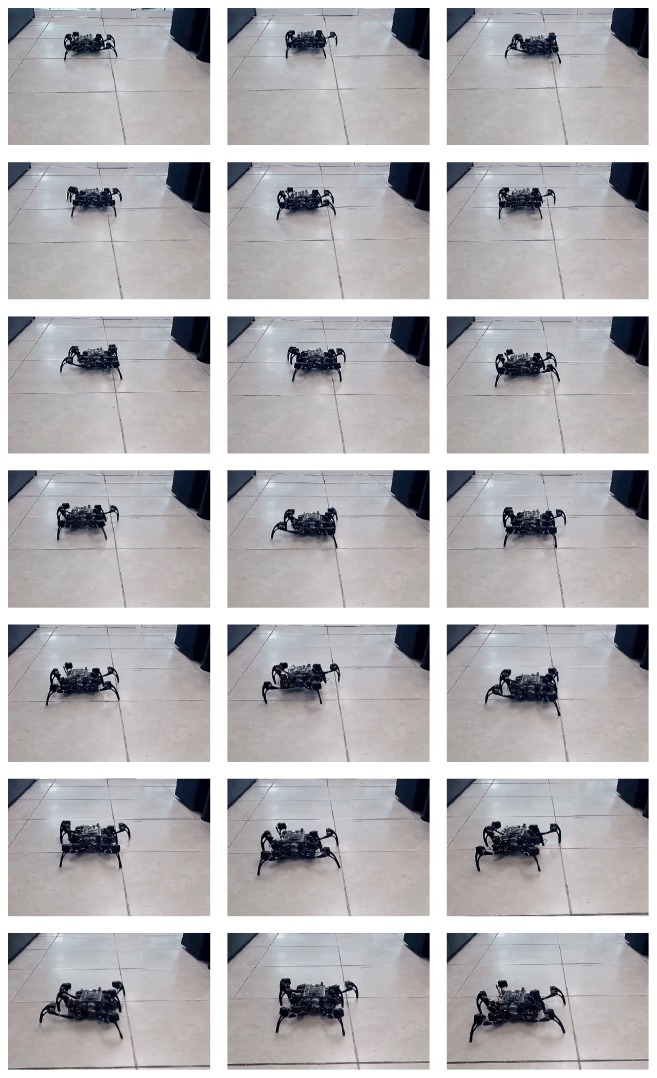
Real time simulation on a hexapod robot with middle legs amputated.

**Figure 11:**
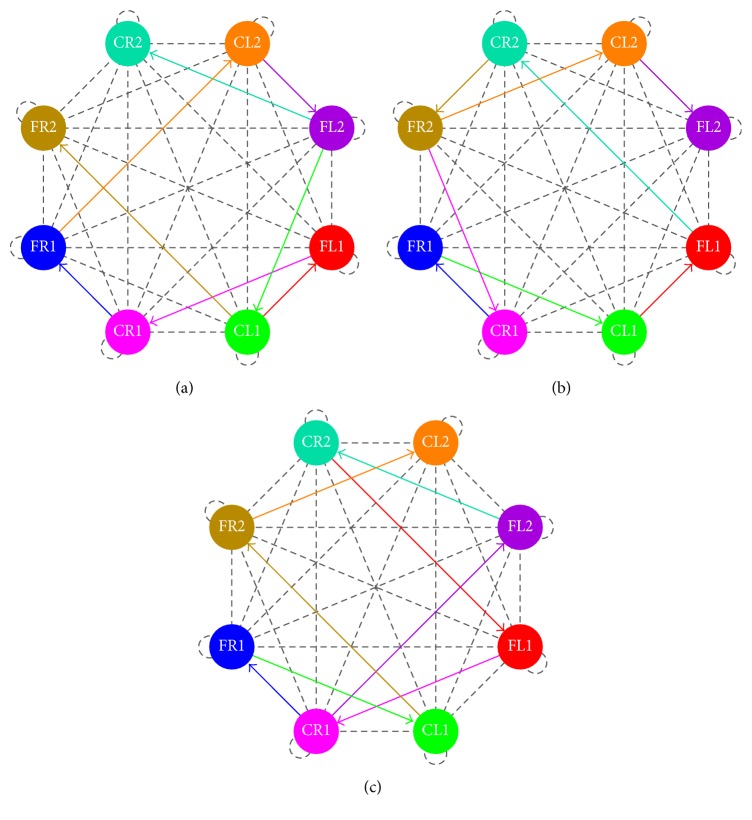
Network topologies with restrictions on the number of synaptic connections for (a) walking, (b) jogging, and (c) running gaits.

**Figure 12:**
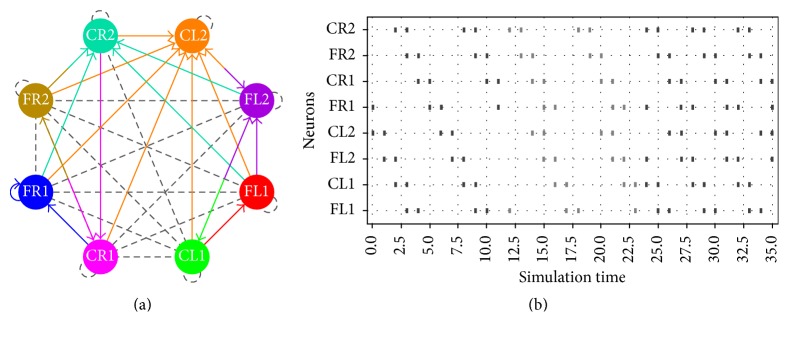
(a) All-in-one topology network. (b) The three locomotion gaits are generated by the same network.

**Figure 13:**
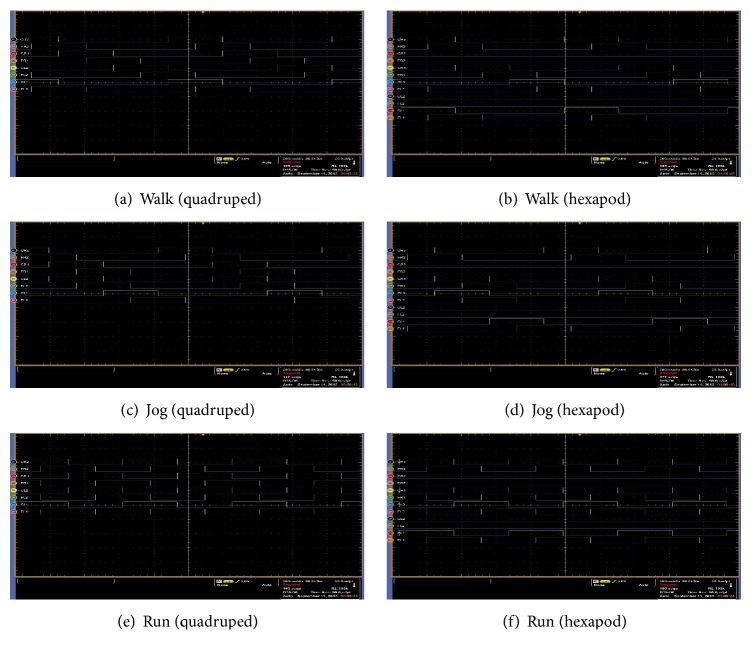
Oscilloscope screenshots for the three locomotion gaits (real time monitoring). On the left side, we show the real time simulation for the quadruped robot, and on the right side the real time simulation for the amputated hexapod robot.

Finally, in Figure 13, we show real time simulations during the walking, jogging, and running gaits of the quadruped and hexapod robots through the use of a digital oscilloscope; in such figure, *x*-axis and *y*-axis represent neuron activity through time and neuron labels, respectively. For the hexapod robot, we can appreciate that two signals are death, because the robot was amputated; for reasons of robot's stability, we have simulated that middle legs were amputated instead of the front legs as in the experimentation reported in [8]. A sequence of the movement of the quadruped and the hexapod robots is shown in Figures 9 and 10, respectively (the sequence in both figures goes from left to right and from top to bottom):

(11) $$\begin{array}{c} w_{aio}=\left|\begin{array}{cccccccc} 0 & 2 & 0 & 0 & 0 & 0 & 0 & 0\\ 0 & 0 & 8 & 0 & 0 & 0 & 0 & 0\\ -1 & -4 & 0 & 7 & 0 & 0 & 0 & 0\\ -3 & 3 & -9 & 0 & 7 & -2 & 6 & -1\\ 0 & 0 & 0 & 0 & -3 & 4 & 0 & 0\\ 0 & 0 & 0 & 0 & 0 & 0 & 9 & -2\\ 0 & 0 & 0 & 0 & 0 & -8 & 0 & 8\\ 5 & 0 & 7 & 0 & -6 & 0 & -5 & 0 \end{array}\right|. \end{array}$$

## 4. Conclusions

A biologically inspired embedded system for quadrupedal robot locomotion has been presented. The design methodology includes a solid mathematical background, biological validation, numerical simulations, and hardware implementation. The theoretical framework includes spiking neurons to reproduce locomotion patterns and a grammar evolution approach to estimate the parameters of such neurons. In this work, we show how the simplest spiking neuron model is able to reproduce periodic patterns such as those observed in living entities. At the same time, we applied the Christiansen Grammar Evolution to estimate the weights and synaptic connections of the spiking neural network to achieve an exact reproduction of the desired locomotion patterns. Also, we have improved the parameter estimation in spiking neural networks by incorporating three different fitness functions and using the SPIKE-distance to compare the effectiveness of each of them.

Numerical simulations have demonstrated the effectiveness of the whole system; however, the major achievement is the hardware implementation of this system on real legged robots. Implementation in high and low level programming languages has been performed in order to control the robots by using Arduino and FPGA boards, respectively.

Real time numerical experiments on two-legged robots (quadruped and hexapod) have been shown in Section 3. This methodology can also be applied to any kind of legged robot. Next step in this research is the use of sensory information for autonomous navigation.

## Figures and Tables

**Algorithm 1:**
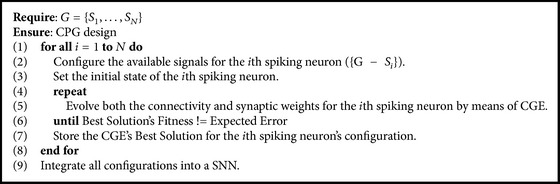
CPG design methodology.

## Appendix

### Christiansen Grammar

A Christiansen Grammar (CG) is a set CG = {Σ_*N*_, Σ_*T*_, *S*, *P*}, where Σ_*N*_ is the set of nonterminals, Σ_*T*_ is the set of terminals, *S* is the initial start symbol (*S* ∈ Σ_*N*_), and *P* is the set of productions (see [19] for more details). Next the CG is shown which allows deriving words that accomplish the restrictions mentioned in Section 2.3 by creating a production which contains a finite number of presynaptic connections (1 ≤ *n* ≤ *N*), besides preventing repetitive connections from a presynaptic neuron by eliminating indexes of presynaptic neurons already connected (id_1_ ≠ id_2_ ≠ ⋯≠id_*n*th_). Next, the herein proposed CG for defining presynaptic connections of a neuron is introduced:

CG = { 
Σ_*N*_ = {〈*neuronSynapses*〉(↓*g*_*i*_), 〈*connections*〉(↓*g*_*i*_, ↑*g*_*o*_),
〈*neuronIdList*〉(↓*g*_*i*_, ↑*n*), 〈*synapses*〉(↓*g*_*i*_), 〈*synapse*〉(↓*g*_*i*_, ↑*g*_*o*_),
〈*weight*〉(↓*g*_*i*_), 〈*sign*〉(↓*g*_*i*_), 〈*digit*〉(*g*_*i*_)},
Σ_*T*_ = {1,2,…, 8,9, +, −, :, |},
*S* = 〈*neuronSynapses*〉(↓*g*_*i*_),
*P* = {〈*neuronSynapses*〉(↓*g*_*i*_)⊨〈*connections*〉(↓*g*_*i*_, ↑*g*_*o*_):
〈*synapses*〉(↓*g*_*o*_){ },
〈*connections*〉(↓*g*_*i*_, ↑*g*_*o*_)⊨〈*neuronIdList*〉(↓*g*_*i*_, ↑*n*){ 
↑*g*_*o*_ = ↓*g*_*i*_ ∪ 〈synapses〉(↓*g*_*i*_)⊨〈synapse〉(↓*g*_*i*_, ↑*g*_*o*_1__) | ⋯
|〈synapse〉(↓*g*_*o*_↑*n*−1__, ↑*g*_*o*_↑*n*__){ }},
〈*neuronIdList*〉(↓*g*_*i*_, ↑1)⊨1{ },
⋮
〈*neuronIdList*〉(↓*g*_*i*_, ↑*N*)⊨*N*{ },
〈*synapse*〉(↓*g*_*i*_, ↑*g*_*o*_)⊨〈*neuronIdList*〉(↓*g*_*i*_, ↑*n*), 〈*weight*〉(↓*g*_*i*_){ 
↑*g*_*o*_ = ↓*g*_*i*_ − {〈neuronIdList〉(↓*g*_*i*_, ↑*n*)⊨  ↑*n*{ }}},
〈*weight*〉(↓*g*_*i*_)⊨〈*sign*〉(↓*g*_*i*_)〈*digit*〉(↓*g*_*i*_){ },
〈*sign*〉(↓*g*_*i*_)⊨+{ },
〈*sign*〉(↓*g*_*i*_)⊨−{ },
〈*digit*〉(↓*g*_*i*_)⊨1{ },
⋮
〈*digit*〉(↓*g*_*i*_)⊨9{ }}
 }
